# Polystyrene Microplastics Induce Oxidative Stress in Mouse Hepatocytes in Relation to Their Size

**DOI:** 10.3390/ijms24087382

**Published:** 2023-04-17

**Authors:** Hui Zou, Huayi Qu, Yusheng Bian, Jian Sun, Tao Wang, Yonggang Ma, Yan Yuan, Jianhong Gu, Jianchun Bian, Zongping Liu

**Affiliations:** 1 College of Veterinary Medicine, Yangzhou University, Yangzhou 225009, China; 2 Jiangsu Co-Innovation Center for Prevention and Control of Important Animal Infectious Diseases and Zoonoses, Yangzhou 225009, China

**Keywords:** microplastics, liver, oxidative stress, SIRT3, SOD2

## Abstract

Microplastics have become a new type of environmental pollutant that can accumulate in various tissues and organs of the body and cause chronic damage. In this study, two different size polystyrene microplastics (PS-MPs, 5 μm and 0.5 μm) exposure models were established in mice to investigate the effects of PS-MPs with different particle sizes on oxidative stress in the liver. The results showed that PS-MPs exposure caused a decrease in body weight and liver-to-body weight. The hematoxylin and eosin staining and transmission electron microscopy results showed that exposure to PS-MPs led to the disorganized cellular structure of liver tissue, nuclear crinkling, and mitochondrial vacuolation. The extent of damage in the 5 μm PS-MP exposure group was more extensive when compared with the other group. The evaluation of oxidative-stress-related indicators showed that PS-MPs exposure exacerbated oxidative stress in hepatocytes, especially in the 5 μm PS-MPs group. The expression of oxidative-stress-related proteins sirtuin 3(SIRT3) and superoxide dismutase (SOD2) was significantly reduced, and the reduction was more pronounced in the 5 μm PS-MPs group. In conclusion, PS-MPs exposure led to oxidative stress in mouse hepatocytes and caused more severe damage in the 5 μm PS-MPs group when compared with the 0.5 μm PS-MPs group.

## 1. Introduction

Microplastics are plastic fragments or particles <5 mm in diameter found in the environment [1]. There has been a sustained increase in global plastic production in recent years. Microplastic pollution has become a global environmental issue due to its small diameter, large relative surface area, and numerous exposure pathways. Exposure to microplastics may occur through ingestion, inhalation, and dermal contact, leading to organ toxicity, neurotoxicity, and reproductive and developmental toxicity. Microplastics can also adsorb other pollutants, including heavy metals and organic pollutants, in the surrounding environment and exert a combined toxic effect. Polystyrene (PS) is one of the most common types of microplastic found in the environment [2].

The human body has a well-established antioxidant defense mechanism, which includes enzymatic and non-enzymatic antioxidant systems. They form a barrier leading to the elimination of reactive oxygen species (ROS) and free radicals generated in the body under normal physiological conditions [3,4]. Oxidative stress is the result of an imbalance between oxidants and antioxidants [5]. Exposure to harmful stimuli leads to the overproduction of ROS, which may be in excess of the capacity of cellular antioxidant defense mechanisms to eliminate it. The excess ROS leads to the formation of oxidized biomolecules, DNA mutation, protein denaturation, and enhanced lipid peroxidation, resulting in oxidative damage to cells, apoptosis, and necrosis [6]. ROS are mainly produced in the mitochondria and endoplasmic reticulum [7]. The liver is a major organ for metabolism and detoxification in mammals and is one of the richest in terms of mitochondrial number and density, making it susceptible to a number of stressors [8]. It is now believed that oxidative stress is a common pathogenic mechanism contributing to several chronic liver diseases. The excessive production or accumulation of ROS in the liver can lead to diseases such as non-alcoholic fatty liver disease (NAFLD) and liver fibrosis [5,9]. Superoxide dismutase (SOD) is an important antioxidant enzyme. According to the different metal cogroups, SOD can be roughly divided into three categories, Cu/Zn-SOD, Mn-SOD, and Fe-SOD [10,11]. Therefore, changes in those trace elements in the body can reflect antioxidant levels on the other hand. Mitochondrial deacetylase sirtuin 3 (SIRT3) is an important regulator of ROS production [7]. Under conditions of stress, it maintains mitochondrial function by deacetylating proteins, specifically superoxide dismutase (SOD2) at lysine 68. This step is essential for maintaining their activities, which regulates ROS and, hence, oxidative stress through the SIRT3/SOD2 pathway [12,13].

Studies have shown that the liver is one of the primary target organs for microplastic accumulation and damage [14,15]. The accumulation of microplastics in the liver can induce excessive production of ROS, leading to oxidative stress in hepatocytes and hepatotoxicity [16]. It has been reported that exposure to microplastics of different sizes has varying effects on liver lipid metabolism in mice, but its effect on oxidative stress in mouse hepatocytes has not yet been definitively established [17]. The present study intends to construct polystyrene microplastics (PS-MPs) injury models, with different sizes of microplastics, in mice, and perform a preliminary study on the effect of polystyrene microspheres of different particle sizes on oxidative stress in mouse hepatocytes.

## 2. Results

### 2.1. Effect of PS-MPs Exposure on Body Weight and Liver Coefficients

**Figure 1:**
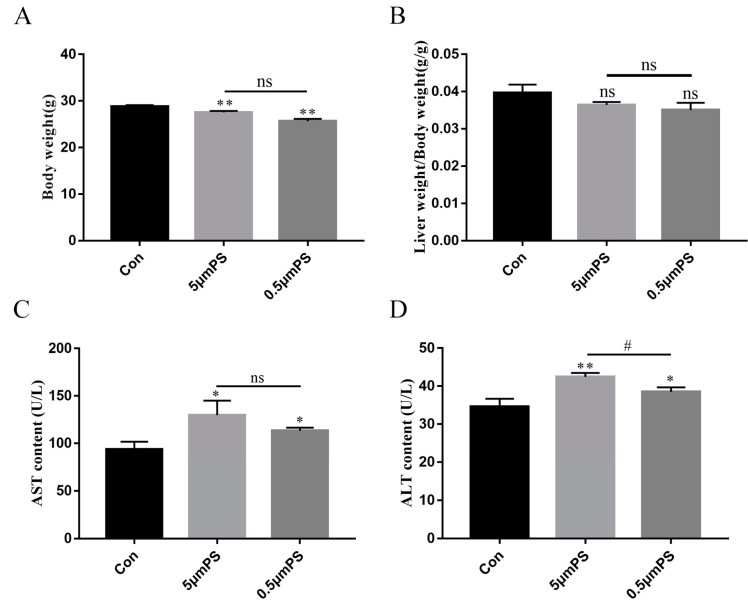
The effect of PS-MPs on body and liver weights. (**A**) Absolute body weight of control and PS-MP-exposed mice. (**B**) Relative uninjured liver wet weight of adult control and PS-MP-exposed mice. (**C**) Serum AST levels in mice. (**D**) Serum ALT levels in mice. Results are shown as mean ± SD (*n* = 3). Compared with the control group, * *p* < 0.05, ** *p* < 0.01. Compared with the 0.5 μm PS-MPs group, # *p* < 0.05. ns indicates not statistically significant.

After 3 months of PS-MP exposure, the body weight and intact liver weight of mice were measured. As shown in Figure 1A, compared with the control group, the body weight of mice treated with 5 μm and 0.5 μm PS-MPs decreased significantly. No significant difference was observed between the body weights of mice belonging to the 5 μm and 0.5 μm PS-MP treatment groups. The ratio of liver weight to body weight showed a decreasing trend, but the difference was not statistically significant (Figure 1B). Compared with the control group, serum AST levels and serum ALT levels in mice treated with 5 μm and 0.5 μm PS-MPs was highly significant or showed a significant increase. No significant difference was observed between the serum AST of mice belonging to the 5 μm and 0.5 μm PS-MP treatment groups. Compared with the 0.5 μm PS-MPs group, the serum ALT of mice belonging to the 5 μm showed a significant increase.

### 2.2. PS-MP-Induced Liver Injury

**Figure 2:**
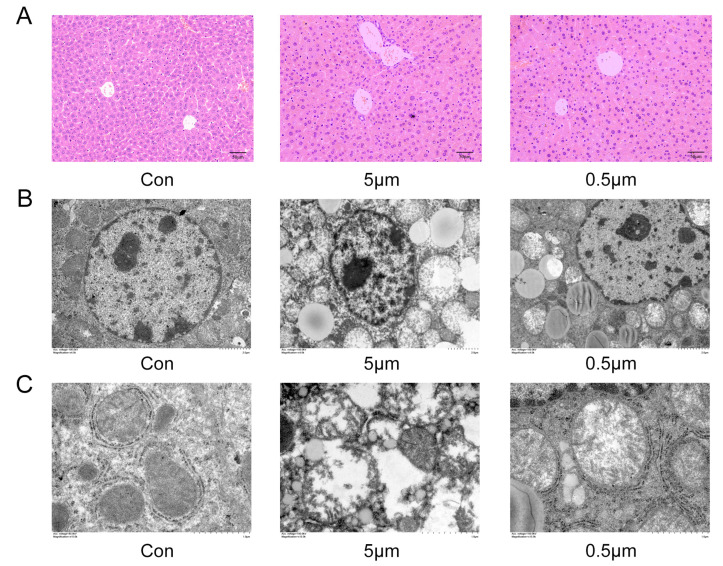
PS-MP-induced liver injury. (**A**) The effect of PS-MPs on liver histopathology was observed by using H&E staining. Scale bar = 50 μm. The effect of PS-MPs on ultrastructure of hepatocytes, observed by TEM. (**B**) The nucleus exposed to PS-MPs of different sizes. (**C**) Mitochondria under exposure to PS-MPs of different sizes. Scale bar = 50 μm. Scale bar in nuclei = 2 μm; scale bar in mitochondria = 1 μm.

The effect of PS-MPs exposure on the histopathology of mouse hepatocytes was observed by using H&E staining. As shown in Figure 2A, in the control group, the hepatocytes were structurally intact, with clear margins, cord-like arrangement, and large, round nuclei. However, in the 5 μm PS-MPs group, the hepatocytes had dilated central venous pores, bruising, swollen cells with indistinct margins, cloudy cytoplasm, and granular degeneration with massive inflammatory cell infiltration. The hepatocytes in the 0.5 μm PS-MPs group were severely swollen with a granular cytoplasm, irregularly arranged, and showed intense nuclear staining.

The structural changes in the mouse hepatocyte nuclei were observed by using TEM. As shown in Figure 2B, in the control group, the nuclei were well defined, and the chromatin was evenly distributed. The nuclei of the cells exposed to 5 μm PS-MPs showed nuclear consolidation, dense staining of nuclear chromatin, and agglutination at the periphery of the nuclear membrane. The nucleus shape changed, and the nuclear pore gap became larger in the group exposed to 0.5 μm PS-MPs. Compared with the 0.5 μm PS-MPs group, the nuclei of the 5 μm PS-MPs group were crinkled to a larger extent. Compared with the control group, the damage to mitochondria due to PS-MPs exposure was equally significant (Figure 2C). In the control group, the mitochondrial structure was intact, the inner and outer membranes were clearly visible, and the mitochondrial cristae were evenly distributed. However, in the 5 μm PS-MP exposure group, the internal structure of mitochondria disappeared into vacuoles. Double-layer membrane fusion was observed, part of which communicated with the cytoplasm, and the mitochondria contained a small number of cristae showing uneven distribution. Mitochondria exposed to 0.5 μm PS-MPs showed swelling, blurring of the bilayer structure, and deformation and loss of cristae. Hence, the mitochondrial damage was more severe in the 5 μm PS-MPs group than in the 0.5 μm PS-MPs group.

**Figure 3:**
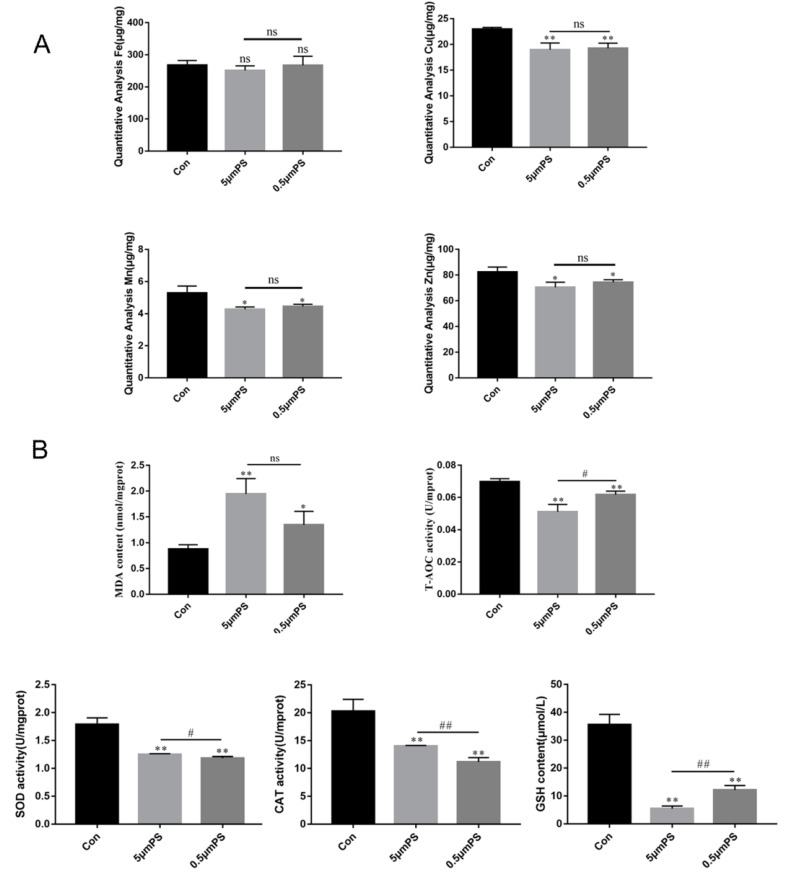
PS-MPs induce oxidative stress in liver. (**A**) Determination of Fe, Cu, Mn, and Zn in mouse liver tissues by AAS. (**B**) Detection of T-AOC, SOD, GSH, CAT activity, and MDA in liver. Results are shown as mean ± SD (*n* = 3). Compared with the control group, * *p* < 0.05, ** *p* < 0.01. Compared with the 0.5 μm PS-MPs group, # *p* < 0.05, ## *p* < 0.01. ns indicates not statistically significant. AAS—atomic absorption spectrometry.

To investigate the effects of exposure to PS-MPs of different sizes on oxidative stress in the liver, the concentrations of trace elements (Fe, Cu, Mn, and Zn) in the liver were determined by using atomic absorption spectrometry. As shown in Figure 3A, no significant difference was observed in the Fe content between the groups. Compared with the control group, treatment with 5 μm and 0.5 μm PS-MPs led to a highly significant decrease in Cu content, while Mn and Zn content also showed significant decreases. Compared with the 0.5 μm PS-MP-treated group, the Cu, Mn, and Zn contents of the 5 μm PS-MP-treated group showed a further decrease, although the difference was not statistically significant. We also measured the levels of MDA and T-AOC and the activities of the antioxidant enzymes SOD, CAT, and GSH. As shown in Figure 3B, compared with control, after 5 μm and 0.5 μm PS-MPs exposure, the activities of SOD, CAT, and GSH in the liver were highly significant. The MDA content was highly significant or showed a significant increase, while the T-AOC enzyme activity showed a highly significant decrease. Compared with the 0.5 μm PS-MP-treated group, the MDA content in the 5 μm PS-MP-treated group was found to further increase, but the difference was not statistically significant, while T-AOC and GSH showed a significant decrease in activity. 

### 2.3. Effect of PS-MPs Exposure on Oxidative-Stress-Related Proteins SIRT3 and SOD2

**Figure 4:**
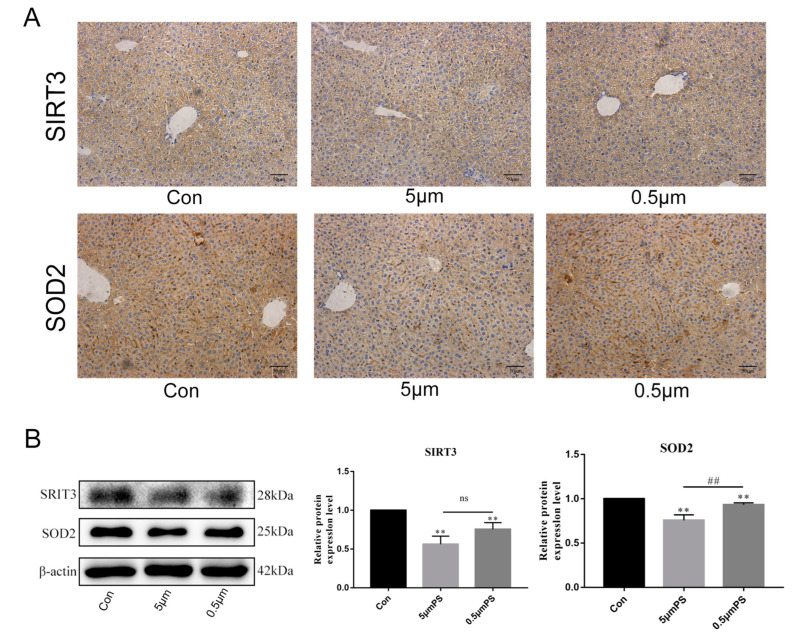
PS-MPs decreased the expression of SIRT3 and SOD2. (**A**) Immunohistochemistry was used to detect the expression of SIRT3 and SOD2 proteins in the liver. Scale bar = 50 μm. (**B**) The levels of SIRT3 and SOD2 were measured using Western blotting, with β-actin serving as an internal control for the cytosolic subfractions. Results are shown as mean ± SD (*n* = 3). Compared with the control group, ** *p* < 0.01. Compared with the 0.5 μm PS-MPs group, ## *p* < 0.01. ns indicates not statistically significant.

To examine the effect of PS-MPs on the expression of oxidative-stress-related proteins SIRT3 and SOD2, the expression levels of the two proteins were examined by using immunohistochemistry and Western blotting. The results of immunohistochemistry are shown in Figure 4A. PS-MPs significantly reduced the expression of SIRT3 and SOD2, with the 5 μm group showing a more pronounced reduction. This result was supported by the Western blot (Figure 4B), which showed a highly significant decrease in the expression of SIRT3 and SOD2 in both treatment groups when compared with the control group. The expressions of SIRT3 and SOD2 showed a highly significant decrease in the 5 μm PS-MP exposure group when compared with the 0.5 μm PS-MP group. 

## 3. Discussion

Global plastic production has increased exponentially in the last few decades, and plastic pollution has emerged as a global threat [18,19]. Plastics may accumulate in the intestines, liver, kidneys, and other organs and lead to dysbiosis of intestinal microbiota, lipid metabolism disorders in the liver, mitochondrial dysfunction in the kidneys, and other toxic effects [4,14,20,21]. After microplastics enter the body’s circulatory system, most of them are transferred to the liver and kidneys and metabolized [22]. Therefore, the liver is one of the most important target organs for microplastics. Microplastics enter the liver through intestinal absorption or epidermal penetration, or through the bloodstream [23]. Studies have shown that the accumulation of microplastics in the liver can induce autophagy and apoptosis of liver cells [24,25]. It can also lead to liver lipid metabolism disorders, aggravate liver fibrosis, and participate in the occurrence of non-alcoholic fatty liver disease (NAFLD) [26,27].

It has been shown that polystyrene microplastic exposure reduces body weight and liver coefficients in mice. The results of this study showed that the mice in the 5 μm and 0.5 μm PS-MPs treatment groups showed a significant decrease in body weight and a decreasing trend in the ratio of liver weight to body weight, which is consistent with the findings of Lu et al. [17]. However, a few studies have shown that PS-MPs exposure has little effect on mouse body weight; the effect of the exposure may be dependent on the size of the microplastic and the duration of exposure [21,28]. Serum biochemical indices are the most widely recognized indices to evaluate liver function [29]. The results of this study showed that both 5 μm and 0.5 μm PS-MPs could cause significant or extremely significant increases in AST and ALT, indicating damage to liver function. This is consistent with the results of Mu et al.’s study on the hepatotoxicity of mice enriched with 5 μm PS-MPs [30]. H&E and TEM studies also demonstrated the damage caused by PS-MPs to the liver and hepatocytes, which included hepatocyte disorganization, nuclear crinkling, and mitochondrial vacuolation.

Oxidative stress is one of the main toxicity mechanisms of microplastics [15]. SOD, CAT, and GSH are important antioxidant enzymes involved in ROS degradation to reduce ROS damage to cells and are an important part of the antioxidant defense mechanism in vivo [4,27]. T-AOC refers to the total antioxidant level composed of various antioxidant substances and antioxidant enzymes, reflecting the total antioxidant capacity of the body [31]. Microplastic exposure causes the overproduction of ROS in the body and activates the antioxidant defense system [32,33]. Wan et al. investigated the changes caused by PS-MPs in the antioxidant enzyme levels of zebrafish larvae and reported an increase in MDA levels and a decrease in the activities of the antioxidant enzymes SOD, CAT, GSH, and T-AOC [34]. This is in agreement with the results of Wang et al. regarding the damage of PS-MPs enrichment in mudskipper larvae [35]. However, Deng et al. constructed a mouse model of PS-MPs exposure and demonstrated an increase in the activities of GSH-Px and SOD, while the CAT activity decreased [15]. These findings are consistent with the observations of Meng et al. who studied the changes in the antioxidant enzyme levels in mice exposed to PS-MPs of different sizes [36]. Yang et al. attempted to evaluate the effect of increasing the exposure time to PS-MPs on the liver tissues of juvenile red crucian carp. The activities of the antioxidant enzymes SOD and GST and MDA levels were found to increase and then decrease with increasing exposure time, while CAT activity showed a wave-like trend of increase and decrease [37]. The antioxidant enzyme levels showed varying results on exposure to PS-MPs. Prokic et al. suggested that this could be related to the size, type, concentration, and exposure time of the MPs, as well as the trophic level of the tissues and organisms under study; the underlying mechanism has not yet been fully elucidated [38]. The study reported that the activities of the antioxidant enzymes SOD, CAT, GSH, and T-AOC were significantly decreased, and the MDA content was significantly increased in the PS-MPs group. Trace element testing showed that Cu, Mn, and Zn, which are trace elements associated with oxidative stress, were significantly decreased. The decrease in the content of Cu, Mn, and Zn, which are coenzyme components of SOD, further indicated that PS-MPs exposure disrupted the oxidative/antioxidative balance of the organism. This led to the accumulation of ROS and aggravated oxidative stress in the liver of mice.

SIRT3 is an important NAD^+^-dependent protein deacetylase found in the mitochondrial matrix and is involved in stress response, cell proliferation, and antiapoptotic processes [39,40]. Numerous studies have shown that SIRT3 can regulate the activity of SOD2 by directly binding to it and deacetylating acetylated SOD2 (As-SOD2) [41,42,43,44,45]. SOD2, a member of the superoxide dismutase family, has an important role in scavenging ROS and the maintenance of ROS homeostasis, thereby making it an essential component in defense against oxidative stress [41,46]. Growing evidence suggests that the SIRT3/SOD2 pathway plays a critical role in neuronal protection, liver health, aging, and carcinogenesis [43,47,48]. Previous studies have shown that toxic stimuli reduce the expression of SIRT3 and SOD2 proteins in the body, resulting in the accumulation of ROS [42,43]. The results of the present study showed that PS-MPs exposure significantly decreased the expression of SIRT3 and SOD2 proteins in the liver, thereby weakening the ability of the body to scavenge ROS and leading to its excessive accumulation in the liver and increased oxidative stress. Further studies are needed to investigate if PS-MPs induce oxidative stress in hepatocytes through the SIRT3/SOD2 pathway.

There are various theories on the correlation between the particle size of microplastics and their toxic effects. Deng et al. concluded that the extent of the accumulation of microplastics in the body is related to their particle size. The smaller the particle size, the greater the accumulation. The toxicity of microplastics is negatively correlated with their particle size [15]. Wang et al. reached similar conclusions that exposure to PS-MPs delayed skeletal muscle regeneration [21]. Hou et al. concluded that there is no direct correlation between the particle size of microplastics and their toxicity or accumulation in the body [28]. Meng et al. also demonstrated that there is no correlation between the size of microplastics and their toxic effects, and that the latter is dependent not only on the adsorption capacity of the target organ but also on the way plastic particles are present in the target organ and the stimulation pathway [36]. Lu et al. constructed exposure models of PS-MPs of different particle sizes (70 nm, 5 μm, and 20 μm) on zebrafish to study their hepatotoxicity. Among the three different sizes of PS-MPs, only the 70 nm and 5 μm PS-MPs could trigger an inflammatory response, oxidative stress, and lipid metabolism disorders in the liver of these fish, and 5 μm PS-MPs caused more severe histopathological changes and more prominent changes in the activities of antioxidant enzymes [25]. The present study involved an investigation into the histopathological and ultrastructural changes in liver samples, analyses of oxidative-stress-related indicators, and a detailed examination of the oxidative-stress-related proteins SIRT3 and SOD2. The results show that exposure to PS-MPs of larger particle size (5 μm) caused more severe liver damage and oxidative stress compared with exposure to smaller PS-MPs (0.5 μm).

In summary, we found that PS-MPs exposure decreased body weight and liver coefficients in mice and caused histopathological and ultrastructural changes in hepatocytes. It significantly reduced the SOD coenzyme Mn, Cu, and Zn content. Additionally, by reducing the activities of antioxidant enzymes SOD, CAT, and GSH, the oxidative/antioxidant balance in the liver was destroyed, the total antioxidant level of the liver was significantly reduced, the lipid peroxidation level was increased, and the oxidative stress of the liver was aggravated. The expression levels of SIRT3 and SOD2 proteins were significantly decreased, which also reflected the decreased ability of the liver to eliminate ROS. Moreover, the results of this study show that exposure to PS-MPs with larger particle size (5 μm) caused more severe liver damage than that caused by exposure to smaller PS-MPs (0.5 μm). This study is a supplement to the field of PS-MPS-induced hepatotoxicity in mice, and the underlying mechanism of PS-MPS-induced mouse hepatocytes needs to be further investigated. 

## 4. Materials and Methods

### 4.1. Animals and Treatment

This study was approved by the Institutional Animal Care and Use Committee of Yangzhou University and carried out in accordance with the Guide for the Care and Use of Laboratory Animals of the National Research Council (approval ID: SYXK (Su) 2017−0044). The dosage and size of PS-MPs were determined according to previous studies [15,20,49]. The two different sizes of PS-MPs (5 µm and 0.5 µm) were purchased from BaseLine ChromTech Research Centre (Tianjin, China). SPF-grade 6-week-old male C57BL/6J mice were provided by Spelford Biotechnology (Beijing, China). After 1 week of pre-rearing at a constant temperature of 22 °C with 12 h of light and 12 h of darkness in a dry and clean environment, the mice were randomly divided into three groups (*n* = 8 each). For drinking purposes, the control group was given double-distilled water, while the animals in the other two groups had double-distilled water containing 5 μm and 0.5 μm PS-MPs, respectively. The PS-MPs concentration in both experimental groups was 10 mg/L. All mice had ad libitum access to feed. After free drinking and feeding for 3 months, the mice were sacrificed by neck dislocation, and their body weights were measured and recorded. The liver tissue was removed, weighed, and immersed in 10% neutral formalin or 2.5% glutaraldehyde for subsequent studies.

### 4.2. Hematoxylin and Eosin (H&E) and Immunohistological Staining

The samples were fixed with paraformaldehyde for 24 h and subsequently trimmed. The fixed tissue was embedded in wax blocks and made into paraffin sections. After a series of gradient dehydration, the paraffin sections were stained with H&E and rinsed with running water. The sections were examined under a microscope (Leica 2500, Leica Corporation, Wetzlar, Germany) and photographed to evaluate the histopathological alterations in the liver. For immunohistochemical staining, microwave antigen repair was used to repair the antigen. Two primary antibodies were used for immunostaining: SIRT3 (1:100, #2627s, Cell Signaling Technology, MA, USA) and SOD-2 (1:50, sc-137254, Santa Cruz, CA, USA), which show bands and quantify positive staining.

### 4.3. Transmission Electron Microscopy (TEM)

The liver tissue was cut into small pieces (1–2 mm) in an ice bath and cleaned with phosphate-buffered saline (PBS), following which they were fixed in fresh 2.5% glutaraldehyde at room temperature for 2 h and transferred to 4 °C for 12 h. The tissues were subsequently fixed in osmic acid, dehydrated in graded ethanol solutions, and embedded in Epon 812 mixture. Ultrathin sections were cut with a diamond knife and negatively stained with aqueous uranyl acetate and lead citrate and observed under a transmission electron microscope (HT-7800, Tokyo, Japan).

### 4.4. Detection of Oxidative Stress-Related Indexes

The major antioxidant enzymes, including superoxide dismutase (SOD), glutathione (GSH), and catalase (CAT), and the oxidative stress indices malonic dialdehyde (MDA) and total antioxidant capacity (T-AOC) were measured using specific commercial kits from the Nanjing Jiancheng Bioengineering Institute (Nanjing, China), according to the manufacturer’s instructions.

### 4.5. Determination of Trace Elements by Atomic Absorption Spectrometry

An adequate amount of sample was taken from each group, dried at 60 °C overnight, and subsequently ground. The dry weight was recorded. The samples were digested using the microwave digestion method and fixed with ultrapure water. The concentrations of selected trace elements (Mn, Cu, Fe, and Zn) in liver tissues were determined by using atomic absorption spectrometry using certified reference material (SRM 1598, NIST).

### 4.6. Western Blotting 

Liver tissue samples (20 mg) were obtained from each group. To these samples, 250 μL of RIPA lysis solution, in which RIPA and protease inhibitor (NCM, Suzhou, China) were configured in a ratio of 1:100, was added, and tissue homogenates were prepared by grinding with an electric grinder. The homogenates were lysed by ultrasonication, centrifuged (12,000 r, 10 min), and the supernatant was collected. The protein concentration was determined using a bicinchoninic acid (BCA) protein assay kit (Beyotime, Shanghai China) prior to normalizing concentrations. Protein samples of the same final volume and concentration were separated by 12% sodium dodecyl sulfate–polyacrylamide gel electrophoresis (SDS-PAGE) and transferred to a polyvinylidene fluoride (PVDF) membrane. The membrane was blocked with 5% skim milk, which was made with TBS-T for 2 h and incubated overnight at 4 °C with the following antibodies: anti-SIRT3 (sc-365175, Santa Cruz, CA, USA, 1:500), anti-SOD2 (sc-137254, Santa Cruz, CA, USA, 1:500), and anti-β-actin (Proteintech, Wuhan, China, 1:1000). The membranes were washed three times with TBST and incubated with HRP-conjugated anti-mouse IgG (Abcam, Cambridge, UK, 1:10,000) at room temperature for 2 h. Subsequently, an enhanced chemiluminescence (ECL) kit (NCM Biotech, Suzhou, China) was used to generate protein bands under a Chemiluminescent Imaging System (Tanon-5200, Shanghai, China). The intensities of the immunoreactive protein bands were quantified using ImageJ software (National Institutes of Health, Bethesda, MD, USA).

### 4.7. Statistical Analysis

The data from at least three independent experiments were analyzed statistically and expressed as mean ± standard deviation (SD). GraphPad Prism 7 software (GraphPad, La Jolla, CA, USA) was used to analyze the data using one-way analysis of variance (ANOVA) (Scheffe’s SF test). A *p*-value of less than 0.05 was considered to be statistically significant, while values less than 0.01 were regarded as highly significant.

## Data Availability

The datasets generated during and/or analyzed during the current study are available from the corresponding author upon reasonable request.
